# Evaluation of standardized doctor's orders as an educational tool for undergraduate medical students: a prospective cohort study

**DOI:** 10.1186/1472-6920-13-97

**Published:** 2013-07-11

**Authors:** Yuna Lee, Ophyr Mourad, Daniel Panisko, Robert Sargeant, Rodrigo B Cavalcanti

**Affiliations:** 1 St. Michael’s Hospital, 4 CCW 149 30 bond st, Toronto, Ontario M5B 1W8, Canada; 2 St. Michael’s Hospital, 30 bond st, Toronto, Ontario M5B 1W8, Canada; 3 Toronto Western Hospital, 399 Bathurst st, Toronto, Ontario M5T 2S8, Canada

## Abstract

**Background:**

Standardized doctor’s orders are replacing traditional order writing in teaching hospitals. The impact of this shift in practice on medical education is unknown. It is possible that preprinted orders interfere with knowledge acquisition and retention by not requiring active decision-making. The objective of the study was to evaluate the impact of standardized admission orders on disease-specific knowledge among undergraduate medical trainees.

**Methods:**

This prospective cohort study enrolled Year 3 (n = 121) and Year 4 (n = 54) medical students at two academic hospitals in Toronto (Ontario, Canada) during their general internal medicine rotation. We used standardized orders for patient admissions for alcohol withdrawal (AW) and for acute exacerbations of chronic obstructive pulmonary disease (AECOPD) as the intervention and manual order writing as the control. Educational outcomes were assessed through end-of-rotation questionnaires assessing disease-specific knowledge of AW and AECOPD.

**Results and discussions:**

Of 175 students, 105 had exposure to patients with alcohol withdrawal during their rotation, and 68 students wrote admission orders. Among these 68 students, 48 used standardized orders (intervention, n = 48) and 20 used manual order writing (control, n = 20). Only 3 students used standardized orders for AECOPD, precluding analysis. There was no significant difference found in mean total score of questionnaires between those who used AW standardized orders and those who did not (11.8 vs. 11.0, p = 0.4). Students who had direct clinical experience had significantly higher mean total scores (11.6 vs. 9.0, p < 0.0001 for AW; 13.8 vs. 12.6, p = 0.02 for AECOPD) compared to students who did not. When corrected for overall knowledge, this difference only persisted for AW.

**Conclusions:**

No significant differences were found in total scores between students who used standardized admission orders and traditional manual order writing. Clinical exposure was associated with increase in disease-specific knowledge.

## Background

Inpatient health-care institutions are increasingly adopting disease-specific order sets [1,2] to decrease practice variation, promote evidence-based practice, improve efficiency [3], decrease the rate of medication errors [4,5] and adhere to practice guidelines [6,7]. Available quantitative research is limited and has been done by few institutions to assess the impact of disease-specific standardized orders. A recent systemic review showed that four academic institutions have demonstrated the efficacy of health information technologies in improving quality and efficiency [3]. The introduction of standardized orders, in both paper and electronic formats, is replacing the traditional method of manually writing orders within academic health sciences hospitals [8]. The impact of this shift in practice on the knowledge acquisition of medical trainees is unknown.

It is unclear whether standardized orders have a positive or negative impact on medical education. Positive impacts may include improvements in disease-specific knowledge, and increased retention of previously acquired knowledge. The information contained in the order sets may also allow for new knowledge acquisition by the trainee (e.g. criteria for use of antibiotics in patients presenting with chronic obstructive pulmonary disease (COPD) exacerbation). Preprinted orders may also improve certainty in the application of previously acquired knowledge. For example, it can provide recommended dosages for benzodiazepine dosage in alcohol withdrawal, or details on specific therapies such as appropriate use of antibiotics for COPD exacerbations. In contrast, it is possible that standardized orders do not require reflection or active decision-making, and therefore, may interfere with knowledge acquisition or retention [9,10]. For example, reliance on a checkbox included in an order set to order deep vein thrombosis (DVT) prophylaxis may result in this particular intervention not being implemented in the absence of the preprinted orders. This issue is concerning if trainees subsequently rotates to a clinical site where the DVT order set is not available. Similarly, knowledge of drug dosages may not be retained because of the lack of the reinforcement provided by writing the dose.

The objective of this study is to assess the impact of standardized orders on medical knowledge acquisition among undergraduate medical trainees. Two standardized order sets were studied, one based on the Clinical Institute Withdrawal Assessment for Alcohol Scale-revised (CIWA-Ar) and a second using acute exacerbation of COPD admissions.

## Methods

### Study subjects

All Year 3 and 4 medical students who were assigned to the General Internal Medicine inpatient Clinical Teaching Unit (CTU) service at two tertiary care hospitals affiliated with the University of Toronto (referred to as Hospital A and Hospital B) between 2007 and 2009 were eligible to participate. Year 3 students were assigned to a CTU for 6 weeks while Year 4 students were assigned for 3 weeks. Students undertook 6 weeks on CTU during their third year clerkship and then rotated again for 3 weeks in their fourth year. Students assigned to Hospital A or B for their third year clerkship remained in the same hospital for their fourth year clerkship. Their duties included assessing patients in the Emergency Department (ED), writing admission orders, and providing care throughout the patients’ admission to hospital. The workload of students at each hospital was very similar. Both third and fourth year students were asked to manage two to three patients at the beginning of their rotation and to increase their workload with up to five to six patients per day towards the end of their rotation. We asked the attending staff and the senior residents to make every effort to adhere to the policy. Both hospitals are large, tertiary inner city hospitals. At Hospital A, 4000 to 4500 patients per year are admitted to the CTU service via the ED, and at Hospital B, 4000–4200 patients per year are similarly admitted. The average number of patients admitted with alcohol withdrawal per year at hospitals A and B are 92 (2% of all admitted cases) and 96 (2% of admitted cases), respectively. The average number of patients admitted with acute exacerbation of COPD per year at hospitals A and B are 200 (4.4%) and 144 (3.4%), respectively. Both hospitals have similar structure and content of educational rounds.

Students were not made aware of the study during their rotation and were invited to participate by writing using end of rotation questionnaires at the end of their CTU rotation.

### Intervention

The CIWA-Ar order set for alcohol withdrawal (AW) and the COPD order set for COPD exacerbation were used at the time of hospital admission at Hospitals A and B, respectively. The use of the CIWA-Ar order set was hospital-based. Trainees at Hospital A used the CIWA-Ar order set at the time of admission, while trainees at Hospital B manually wrote admission orders based on their own knowledge and any other tools available to them in the management of alcohol withdrawal symptoms. For patients with COPD exacerbation, the COPD order set was used at Hospital B at the time of admission, while manual order writing was done at Hospital A.

Both hospitals have developed several standardized order sets. Among them, CIWA-Ar and COPD order sets were selected because they are unique to each participating hospital. The CIWA-Ar order set has been developed by a pharmacist and a general internist and implemented only at Hospital A. Only Hospital A trainees have access to the CIWA-Ar order set. The CIWA-Ar order set has been used since November 2006. In contrast, the COPD order set has been implemented only at Hospital B, and only Hospital B trainees have access to the COPD order set. This order set has been in clinical use since July 2006. Both order sets have been approved by their appropriate hospital committees. CIWA-Ar was also selected to assess whether the educational impact of standardized orders have beneficial educational effects since undergraduate trainees are less familiar with CIWA-Ar at the beginning of their training. This lack of familiarity would maximize the relative differences between order entry formats.

### Outcome measures

Disease-specific knowledge was assessed by end of rotation questionnaires that covered knowledge in AW and COPD. These questionnaires were administered only at the end of the rotation. A pre-test was not administered since this may have altered the result of the end of rotation questionnaires due to the potential ability of students being aware of the questions based on the pre-test. Questionnaires were developed by two general internists involved in the study, and reviewed by three other general internists, who have expertise in medical education and the management of pulmonary disease and drug intoxication. They were designed to gather information on the use of standardized orders and specific content knowledge relating to the management of patients with alcohol withdrawal or COPD exacerbation. The questionnaires were comprised of multiple choice, short answer and true/false questions (Additional file 1). Students were also asked to write admission orders based on a clinical vignette, and to specify the dose and frequency of medications. The multiple choice questions, true or false questions and short answer questions were designed to assess the specific content knowledge relating to these two medical conditions which are included in the order sets. For example, in the CIWA-Ar order set, physicians are reminded of contraindications and precautions before a benzodiazepam is ordered. Also, physicians are asked to order thiamine and multivitamin for a patient with alcohol withdrawal. In case of the COPD order set, physicians are reminded to assess for smoking cessation and the indications for the use of antibiotics and systemic steroids for a patient with COPD exacerbation. In addition to the end of rotation questionnaires, students were asked about the previous exposure to writing an admission order for COPD or alcohol withdrawal and the previous exposure to the CIWA-Ar order set or the COPD order set.

Questionnaires were graded by a research assistant and based on a marking scheme (Additional file 1) developed a priori by two of the authors (YL, RBC). Two authors (YL, RBC) also independently marked the whole test. If there were any discrepancies in the total exam score, the discrepancy was resolved by the Principal Investigator (PI). The research assistant and two authors were not formally blinded, but the group assignments were not readily available when marking the tests.

The primary outcome measure was the total scores. Total scores was the score on the AW or AECOPD sections including the score of MC questions, the score of short answer questions and the score of order writing question. The secondary outcome measure was the score on the admission order writing portion of the questionnaires.

### Data analysis

The unit of analysis was the individual student. The intervention group was defined as students who wrote admission orders using standardized order sets for each condition during their rotation, compared to students who wrote orders without using standardized orders. For the primary analysis, total scores of the questionnaires were compared between groups using t-tests. Multivariable regression models were used to assess the impact of use of standardized orders on questionnaire scores when controlled for year of training (Year 3 vs 4), base hospital (Hospital A vs B), as well as overall student knowledge assessed by the internal medicine end-of-rotation final written exam. Participants with missing data were excluded from the analyses.

Given the potential impact of exposure to a clinical case on learning without necessarily writing orders, a preplanned comparison was performed based on clinical exposure to the condition. The questionnaire scores for each of the conditions (AW and AECOPD) were compared between students who were exposed to a clinical case during their rotation and those who were not, using student’s t tests.

To assess the validity of our outcome measure, Pearson correlation coefficients were calculated between the total scores of the questionnaires and the end-of-rotation final written exam scores.

Based on an expected difference of 15% (70 vs. 85%) on the total scores in the outcome measure questionnaires, we estimated that 120 participants were needed to achieve 80% power in detecting a difference at a = 0.05 with a two-tailed t-test for the primary analysis.

This study was reviewed and received approval from the Research Ethics Boards at the University of Toronto and at both hospitals.

## Results

**Figure 1:**
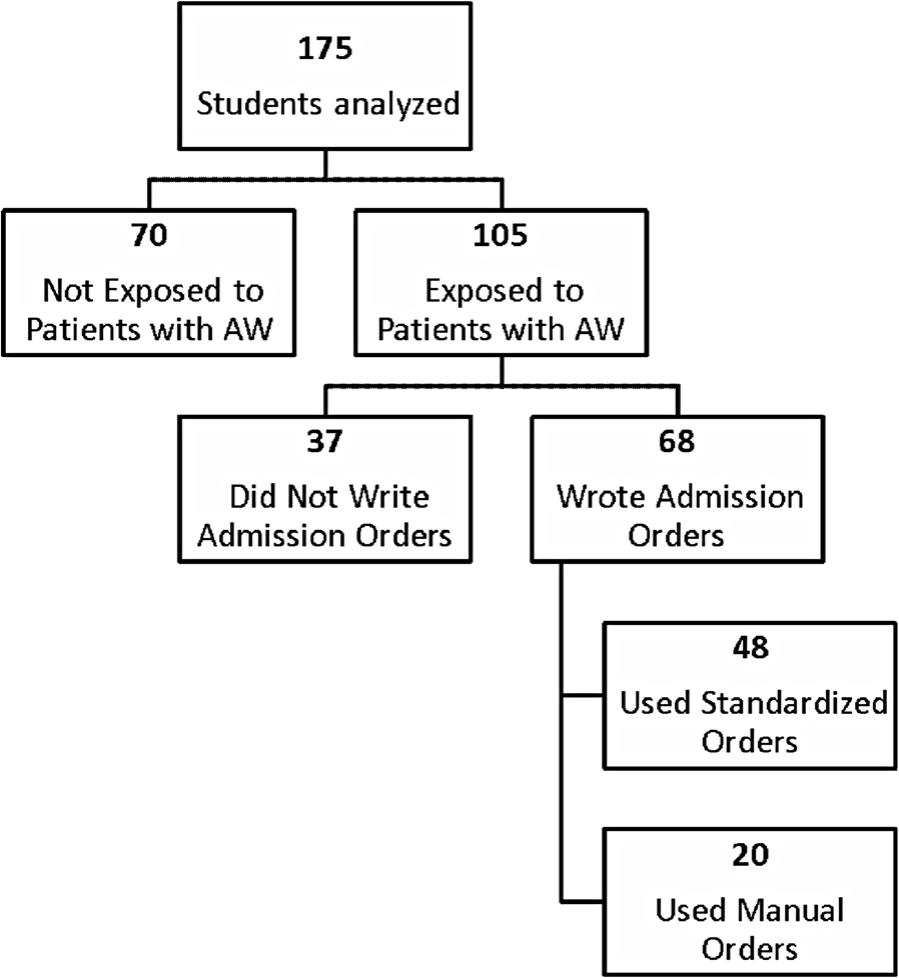
Flow sheet of students analyzed, exposed to patients with AW, wrote admission and exposed to standardized orders for AW.

**Figure 2:**
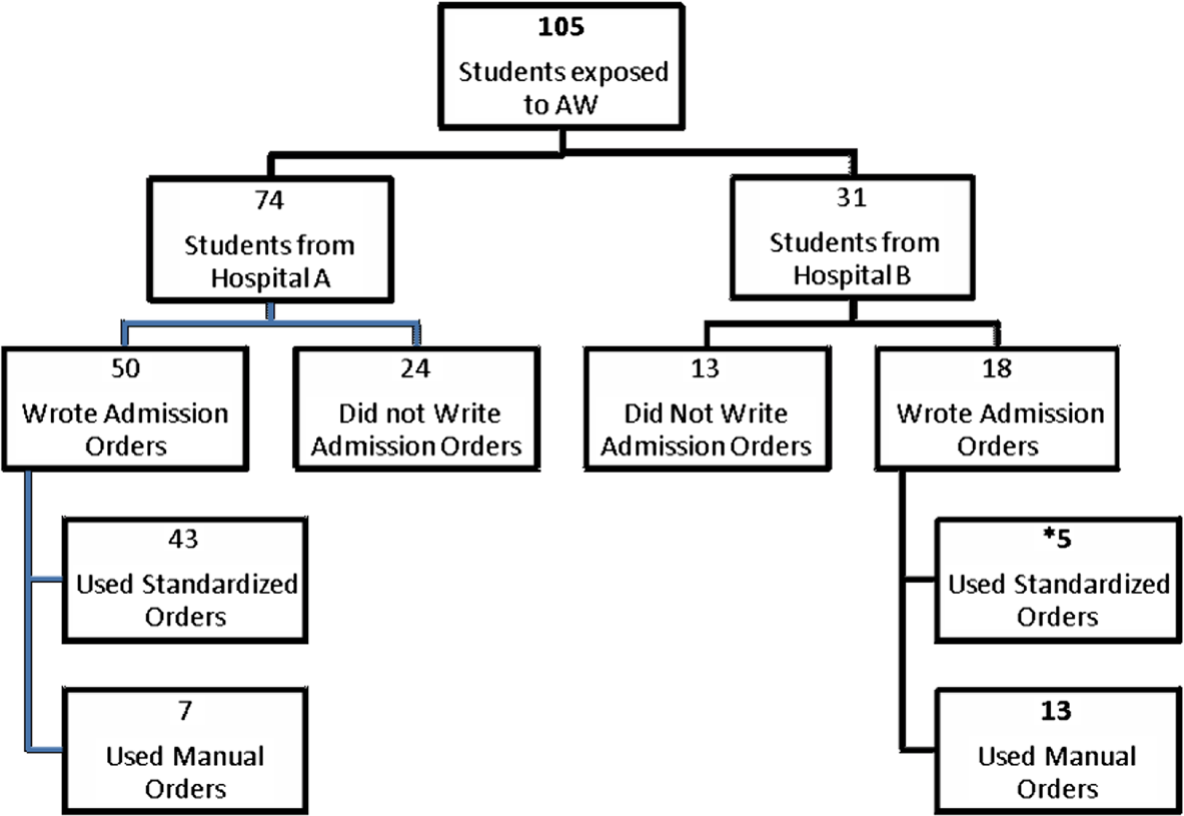
**Flow sheet of students exposed to AW and use of CIWA-Ar orders by students from each hospital.** *Student were identified as admitted to using order set at other hospital which were obtained from past exposure from previous elective rotations.

Out of 201 eligible students, 176 students (84%) agreed to participate in the study. For the AW condition, 175 had complete data for analysis. One hundred twenty one students were year 3 and 54 students were year 4 students. Of 175 students who completed the AW questionnaires, 105 students were exposed to patients with AW, and among them 68 wrote admission orders during their Team Medicine rotation. Of these 68 students, 48 used the standardized admission orders (intervention, n = 48) while 20 students wrote admission orders manually (control, n = 20) (Figure 1). Out of 105 students exposed to patients with AW, 74 students were from Hospital A and 31 students were from Hospital B. Forty three students out of 50 students from Hospital A when admitted patients with AW used standardized orders while 5 students out of 18 from Hospital B used standardized orders when they admitted patients with AW (Figure 2).

**Figure 3:**
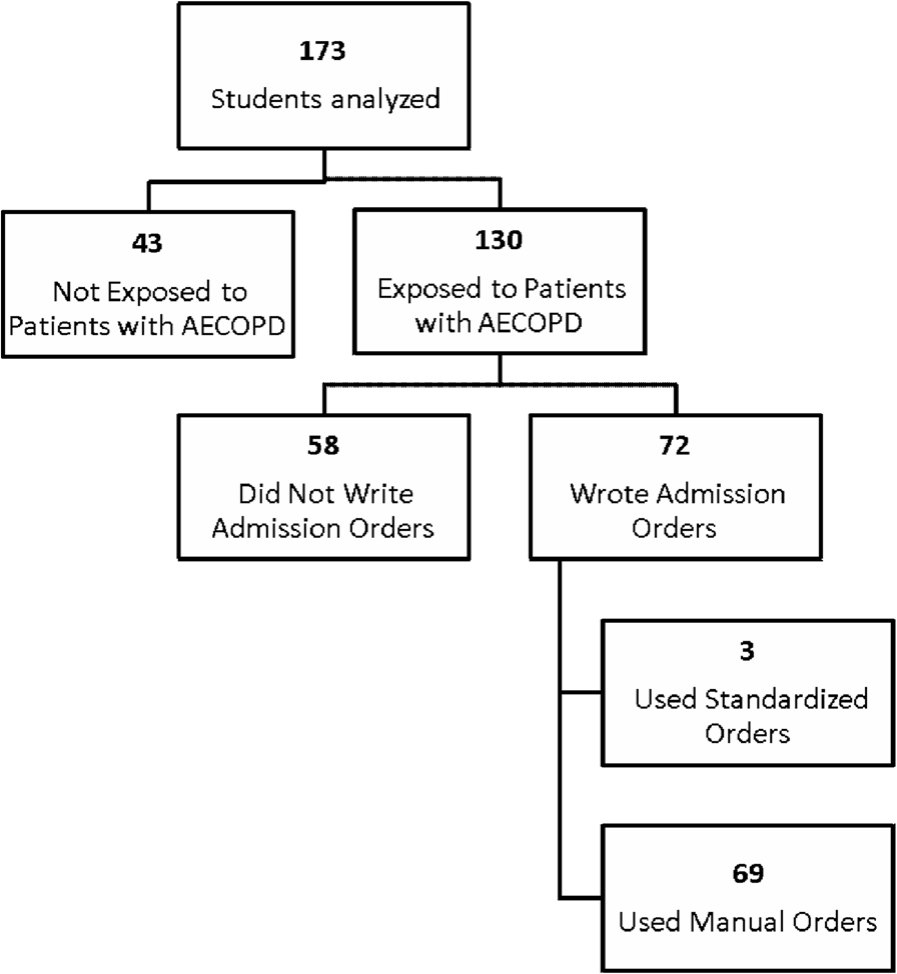
Flow sheet of students analyzed, exposed to AECOPD, wrote admission orders and exposed to standardized doctor’s orders for AECOPD.

For the AECOPD condition, of 173 students who completed the COPD questionnaires, 130 were exposed to patients with AECOPD, and among them, 72 students wrote admission orders. Of the 72 students, only 3 used standardized orders and 69 students wrote admission orders manually (Figure 3). Low rates of standardized order set use for AECOPD precluded further analysis, and therefore, they were excluded from further analysis.

To assess the agreement between the faculty members in grading the admission orders and questionnaires, we calculated the kappa value between them. Total scores of CIWA-Ar order sets had a kappa value of 0.61, and the scores on the admission order writing portion of the questionnaires had a value of 0.55. Total scores of AECOPD order sets had a kappa value of 0.63 and the scores on the admission order writing portion had a value of 0.59.

To determine whether standardized orders affected total scores of the questionnaires, we compared total scores between students who used the CIWA-Ar order sets and those who did not. On univariate analysis, there was no significant difference in the primary and secondary outcome measures (mean score of 11.8 vs 11.0, SD = 3.51, p = 0.4, CI −2.66, 1.04 and 4.2 vs 3.7, SD = 2.10, p = 0.2, CI −1.57, 0.65 respectively). The multivariate analysis similarly did not show significant differences in total scores of the questionnaire between those that used order sets and those who did not (covariate parameter estimate −0.41 95% CI −2.44, 1.62) (Table 1). There was no significant difference in the secondary outcome measure (the score on the admission order writing portion of the questionnaires), between those who used order sets and those who did not (covariate parameter estimate −0.14, 95% CI −1.32, 1.03).

**Table 1:** Multivariate analysis

**Parameter**	**Estimate**	**Confidence interval**	**Pr > ChiSq**
Use of CIWA-Ar order set Vs. Those who used manual order writing	−0.41	−2.44 to1.62	0.69
Year 3 students vs. Year 4 students	−1.20	−2.92 to 0.52	0.17
Base hospital A vs B	0.49	−1.63 to 2.61	0.65
Written exam	0.12	0.02 to 0.22	0.01

Analysis of Parameter Estimates.

Baseline variables: Year 3 vs 4 students, Base Hospital A vs B and End of Rotation Final Written exam.

Total scores for AW and AECOPD questionnaires were modestly positively correlated with students’ marks on the end-of-rotation final written exam (Pearson correlation of 0.22, p < 0.0001 for CIWA, 0.28, p < 0.0001 for AECOPD). Further, the total scores were positively correlated with level of training. The total mean scores of AW questionnaires for Year 3 and Year 4 were 9.73 and 12.36 respectively (P < 0.0001).

To examine whether clinical exposure of AW and AECOPD improved students’ knowledge, we compared the questionnaire scores between students who did and did not report clinical exposure to AW and AECOPD. Students reporting clinical exposure had significantly higher total mean scores for both AW (11.6 vs. 9.0 p < 0.0001, difference of 2.59, CI 1.49, 3.70) and AECOPD (13.8 vs. 12.6 p = 0.02, difference of 1.14, CI 0.03, 2.25). The students’ end-of-rotation final written exam marks were used as an overall control for general knowledge in internal medicine. When corrected for overall knowledge by using the students’ final written exam, this difference persisted for AW (score difference 2.60, 95% CI 1.59, 3.62) but not for AECOPD (score difference 0.83, 95% CI −0.17, 1.83).

## Discussion

This study is the first quantitative study to report the effect of using standardized orders on medical knowledge of specific disease conditions for knowledge acquisition. There were questions regarding the dose of thiamine, multivitamins and benzodiazepam. It is possible that students who used manual order writing may learn and remember the dose of the medication better while the students who used the standardized admission orders may not be able to retain the information on the dose of the medication due to the lack of reflection.

Our study failed to detect a significant difference in total scores between students who used CIWA-Ar order sets and those who did not. Thus, it is possible that the use of standardized orders may not affect disease-specific knowledge acquisition among medical students. However, larger, well-powered studies are clearly required.

If confirmed, there appears to be no negative impact of standardized orders on medical knowledge acquisition despite the potential concerns with lack of reflection or active decision-making by using standardized order sets. Our study results are different from the previous qualitative study performed by Ash et al. This study surveyed residents regarding their perception of computer orders using focus group and individual interviews. It identified concerns by trainees that standardized orders may negatively impact knowledge acquisition from the lack of thoughtful reflection [9]. A similar study conducted by the same group described similar concerns about the use of computer order entry systems at teaching hospitals [10].

Our study adds important information by quantitating the educational impact of standardized orders on disease-specific knowledge and by controlling the results for students’ general knowledge in internal medicine. The questionnaires of AW and AECOPD knowledge were reviewed by practicing internists and were positively correlated with the final written exam scores, indicating criterion validity for the outcome measure. Despite the subjective nature of the admission orders, and the variability of student responses, the kappa values between the faculty members in grading the admission orders and questionnaires reflected moderate to substantial agreement [11].

Another key finding of our study was that clinical exposure was associated with an increase in disease-specific knowledge, particularly for AW. The positive effect of having clinical exposure to AW remained significant when corrected for overall knowledge whereas the effect on AECOPD remained positive but was non-significant. Since the medical school teaching at our university emphasizes COPD more than substance withdrawal, these results may indicate a net benefit of clinical exposure on knowledge acquisition for clinical entities incompletely covered in formal curricula.

Our study has several limitations. First, test raters were not formally blinded to the exposure of students to intervention. To address this problem, we based our marks on an a priori defined marking scheme, and group assignment was not readily available when marking the tests.

Second, it is possible that as the study progressed, students became aware of the study question and focused their learning on AW and AECOPD. This could potentially have attenuated any positive impact of order sets on disease specific knowledge acquisition. However, when tested, there was no significant difference in scores as the study progressed. As a result, the impact of order sets most likely was not attenuated.

Third, the use of the COPD order set was very low (7%), likely because the AECOPD standardized orders were only accessible by a computerized entry system, while the AW pre-printed orders were more readily accessible as paper forms. Students’ lack of familiarity with the computerized order-entry system may have contributed to decreased use of AECOPD orders. This finding of low adaption rate was observed in previous studies which was thought to be due to technical/implementation issues including usability, time, training and frequent deactivation of the system as well as cultural and behavioral issues [10,12,13]. The usability of a computerized physician order entry (CPOE) system was found to play a significant role in its acceptance. One study by Chan suggested a user centered design CPOE prototype to increase its acceptance [14,15].

Fourth, student allocation was opportunistic, based on their assignment to each hospital. To assess the potential for selection bias, we reviewed average scores of student’s medicine final written exam for each hospital A and B. There was no difference in the average scores of the students’ final written exam in Medicine between students based at Hospital A and Hospital B. This finding suggests there was no significant difference in general knowledge of internal medicine between the control and intervention groups.

Fifth, both hospital A and B are similar in the number of patients admitted to the CTU services via the ED, in the average number of patients admitted with AW and COPD exacerbation per year, and in how they offer similar standardized educational curricula for students. These similarities helped minimize possible confounding.

Sixth, there were 5 students from Hospital B who used CIWA-Ar order set only available at Hospital A. These students had the exposure to the order set through their elective rotation at Hospital A. Even though those students were analyzed as part of intervention group, the contamination of the results should be minimal because of the similarities between the two hospitals and no significant difference in average scores of students’ final written exam in Medicine between students based at Hospital A and Hospital B.

Seventh, given that the exposure to clinical cases was self-reported, students who remembered seeing and admitting cases of AW and COPD may be systematically different from students who did not recall. This is one of the confounders in this study. This systematic difference could have positively affected the association of clinical exposure with an increase in disease-specific knowledge.

Eighth, Year 3 and 4 students most likely have differences in exposures and experiences. Combining them into the same groups and testing them on a knowledge based questionnaires could introduce significant heterogeneity to the results. When we reviewed the distribution of year 3 and 4 students in the control and intervention groups, there were 20% of fourth year students (out of 20) in the control group and 28% of fourth year students (out of 46) in the intervention group for the CIWA order set. The slight increase in proportion of fourth year students in the intervention group could have increased the total score resulting in no difference in total scores between both groups. It is possible that the intervention group scores would be lower if the proportion of fourth year students were equal between comparison groups.

Finally, despite recruiting 176 students, the low number of students who wrote admission orders caused our study to be significantly underpowered and we failed to reject the null hypothesis. Given this limitation, it is difficult to generalize the effect of standardized order set on the medical students’ knowledge and a larger study from other hospital systems is required to assess the impact of order sets and generalizability.

## Conclusion

This study did not detect a significant difference in total scores between students who used standardized order sets compared to those who did not, but did show that exposure to clinical cases was associated with improvement in students’ disease specific knowledge. Educators should remain attentive to maximize opportunities for clinical exposure on clerkship rotations, especially for clinical entities that are not commonly included in formal curricula.

## Competing interests

None of the authors have financial conflicts of interest.

## Authors’ contributions

YL: designed the study, analyzed and interpreted the data and drafted the manuscript and finished the final version. RC: participated in designing the study, analyzing and interpreting the data and wrote parts of the manuscripts. DP: made contributions to conception of data and revising the manuscript. RS: helped with ethics approvals and revising the manuscript. OM: helped design the study and revised critically for important intellectual content. All authors read and approved the final manuscript.

## Pre-publication history

The pre-publication history for this paper can be accessed here:

http://www.biomedcentral.com/1472-6920/13/97/prepub

## Supplementary Material

Additional file 1 Order Set Educational Research. Click here for file
