# Supplementary material for: Evaluation of standardized doctor's orders as an educational tool for undergraduate medical students: a prospective cohort study
Source: BMC Med Educ. 2013 Jul 11;13:97. doi: 10.1186/1472-6920-13-97 (PMC3710495; doi:10.1186/1472-6920-13-97)
Supplement: Additional file 1 — Order Set Educational Research. [file 1472-6920-13-97-S1.pdf]

Appendix 1

ORDER SET EDUCATIONAL RESEARCH **Survey #:**

The results of the test will be used ONLY for research purposes and will be anonymous and confidential.

Date: / /

1) Where did you do your current Team Medicine?

SMH____ TWH____

2) What is the level of your training?

CC3____ CC4_____

CIWA-Ar ORDER SET STUDY QUESTIONS **Survey #:**

Check the box that best describes your clinical experience in the past 24 months with a patient whose most responsible admission diagnosis was alcohol withdrawal:

❑I saw the patient in the emergency department and participated in writing of the admission orders for alcohol withdrawal

If you have written the admission orders, please check one of the boxes below.

❑I have used the Clinical Institute Withdrawal Assessment for Alcohol Revised Scale (CIWA-Ar) order set for the admission orders.

❑I have NOT used the CIWA-Ar order set for the admission orders.

❑I was involved in the care of a patient with alcohol withdrawal but did not write admission orders

❑I had no direct clinical exposure to a patient with alcohol withdrawal

Section 1: Multiple Choice Section – select a single correct answer

A) Which one of the following is not a contraindication for using benzodiazepines? ___

a. acute narrow angle glaucoma

b. renal failure

c. hypersensitivity to diazepam or lorazepam

d. coma

TOTAL SCORE: _____/2

B) Which medication is better to be used in a patient with alcohol withdrawal and end stage liver disease? ____

a. Diazepam

b. Lorazepam

c. all of the above

d. none of the above

TOTAL SCORE: _____/2

Section 2: Provide as many answers as possible:

A) What additional medications would you prescribe on admission for a patient with alcohol withdrawal in addition to Benzodiazepines? Please list two medications.

____

____

TOTAL SCORE: _____/

B) List the 7 clinical parameters you would use to monitor the severity of alcohol withdrawal to make decisions about benzodiazepine administration.

____

____

____

____

____

____

____

____

TOTAL SCORE: _____/7

Section 3:

Case: A 25 year old man with a history of alcoholism presents to the emergency department with confusion, tremor and palpitation. His past medical history is not significant. He is not on any medication. On physical exam, he is arousable but disoriented. His heart rate is 105/min, regular with a blood pressure of 140/90mmHg, a respiratory rate of 14/min, temperature of 37.1 degree C, and O2 saturation of 95% on room air. His face is slightly flushed. His thyroid exam is normal. His JVP is flat. The rest of cardiac, respiratory, abdominal, and neurologic examination is unremarkable. He has resting tremor in his hands. Initial lab investigations show: Hb 125g/L with MCV 100, normal WBC and platelet counts, normal electrolytes and creatinine. TSH is also normal. Serum alcohol level is elevated.

Write admission orders for the patient presented in the clinical vignette: (please specify the dose/frequency when medication(s) is (are) ordered).

TOTAL SCORE: _____/10

<u>TOTAL SCORE: _____/23</u>

Survey #:

COPD ORDER SET STUDY QUESTIONS

Check the box that best describes your clinical experience in the past 24 months with a patient whose most responsible admission diagnosis was COPD exacerbation:

❑I saw the patient in the emergency department and participated in writing of the admission orders for COPD exacerbation

If you have written the admission orders, please check one of the boxes below.

❑I have used the COPD order set for the admission orders.

❑I have NOT used the COPD order set for the admission orders.

❑I was involved in the care of a patient with COPD but did not write admission orders

❑I had no direct clinical exposure to a patient with COPD

Section 1: Provide as many answers as possible:

A.Antibiotics have been shown to have a beneficial effect in COPD patients who present with….(list 5 features)

____

____

____

____

____

TOTAL SCORE: ____/5

Section 2: Multiple Choice Section – select a single correct answer

A.Non-invasive positive pressure ventilation has been shown to reduce mortality in acute exacerbations of COPD. The number needed to treat to prevent one death is:

a.4

b.10

c.20

d.70

TOTAL SCORE _____/2

B.Which one of the following is NOT a risk factor for treatment failure:

a.CHF

b.FEV1 < 50% predicted

c.Obesity

d.> 4 exacerbations per year

TOTAL SCORE _____/2

Section 3: TRUE or FLASE

A.Smoking cessation can reduce the rates of exacerbations by 1/3

TOTAL SCORE: _____/2

B.Smoking cessation slows decline in FEV1 in patients with COPD

TOTAL SCORE: _____/2

Section 4:

Case:

A 65-year-old former heavy smoker with a history of chronic obstructive pulmonary disease (COPD) presents to the emergency room with a four-day history of increasing dyspnea. He also reports an increase in volume and purulence of phlegm. His vitals are: heart rate 96, RR 24, BP 140/60, T 36.7 deg Celsius. Oxygen saturation is 85% on room air and improves to 92% with 2L NP oxygen. Chest X-Ray shows hyperinflation but no infiltrates. Arterial blood gases reveal hypoxemia (pO2 60 mmHg) and a mild acute respiratory acidosis.

Write admission orders for the patient presented in the clinical vignette (please specify the dose/frequency when medication(s) is (are) ordered):

TOTAL SCORE: _____/10

TOTAL SCORE: _____/23

APPENDIX 2

Section 1: Multiple Choice Section – select a single correct answer

A) Which one of the following is not a contraindication for using benzodiazepines? ___

a. acute narrow angle glaucoma
b. renal failure
c. hypersensitity to diazepam or lorazepam
d. coma

TOTAL SCORE: _____/2

B) Which medication is better to be used in a patient with alcohol withdrawal and end stage liver disease? ____

a. Diazepam
b. Lorazepam
c. all of the above
d. none of the above

TOTAL SCORE: _____/2

Section 2: Provide as many answers as possible:

A) What additional medications would you prescribe on admission for a patient with alcohol withdrawal in addition to a Benzodiazepines? Please list two medications.

____
____

TOTAL SCORE: _____/2
Ans: MVT, Thiamine, give one mark for MVT or Thiamine. Give two marks for both

B) List the 7 clinical parameters you would use to monitor the severity of alcohol withdrawal to make decisions about benzodiazepine administration.

____
____
____
____
____
____
____
____

TOTAL SCORE: _____/7

Ans:
One mark for Nausea or vomiting
One mark for Tremor
One mark for Paroxysmal sweats
One mark for Anxiety or Agitation
One mark for Tactile disturbances, Auditory disturbances or Visual disturbances
One mark for Headache
One mark for Orientation or clouding of sensorium

Section 3:

Case: A 25 year old man with a history of alcoholism presents to the emergency department with confusion, tremor and palpitation. His past medical history is not significant. He is not on any medication. On physical exam, he is arousable but disoriented. His heart rate is 105/min, regular with a blood pressure of 140/90mmHg, a respiratory rate of 14/min, temperature of 37.1 degree C, and O2 saturation of 95% on room air. His face is slightly flushed. His thyroid exam is normal. His JVP is flat. The rest of cardiac, respiratory, abdominal, and neurologic examination is unremarkable. He has resting tremor in his hands. Initial lab investigations show: Hb 125g/L with MCV 100, normal WBC and platelet counts, normal electrolytes and creatinine. TSH is also normal. Serum alcohol level is elevated.

Write admission orders for the patient presented in the clinical vignette: (please specify the dose if medication(s) is (are) ordered).

Admit to medicine
Diet as tolerated (DAT)
Activity as tolerated (AAT)

Vital signs more frequently than every 12 hrs. at least every 6h (**1 point)**

IV at least 50 cc/hr can be either NS or 2/3 1/3, no D5W given flat JVP **(1 point)**

ECG given tachycardia **(1 point)**

LFT (AST, ALT, ALP, bilirubin) **(1 point)**

INR, PTT - **(1 point)**

Urine/serum tox screen, osmolality **(0.5 point for urine/serum tox screen, 0.5 point for osmolality**

MVT (multivitamin) 100 mg od *3d **(0.5 point for each drug name 0.5 point for each dose)**

Thiamine 100 mg od * 3 d **(0.5 point for each drug name 0.5 point for each dose)**

Choose Valium or Ativan for CIWA scale **(1 point for choosing valium or ativan)**

If CIWA less than 10, no meds **(0.5 point for assessing whether patient needs valium or ativan depending on the CIWA score)**

If CIWA b/w 10-20, give valium (5-15 mg) or ativan (1-3mg)
If CIWA more than 20, give valium (20-40mg) or ativan (3-4mg) mg and do more frequent vital signs
(0.5 point for dose)

TOTAL SCORE: _____/10

Section 1: Provide as many answers as possible:

A. Antibiotics have been shown to have a beneficial effect in COPD patients who present with….(list 5 features)

Answers: An increase in all three of the following cardinal symptoms: dyspnea, sputum volume, and sputum purulence, fever, infiltrate on CXR

TOTAL SCORE: ____/5

Section 2: Multiple Choice Section – select a single correct answer

A. Non-invasive positive pressure ventilation has been shown to reduce mortality in acute exacerbations of COPD. The number needed to treat to prevent one death is:

a. 10

TOTAL SCORE _____/2

B. Which one of the following is NOT a risk factor for treatment failure:

a. CHF
b. FEV1 < 50% predicted
c. Obesity
d. > 4 exacerbations per year

TOTAL SCORE _____/2

Section 3: TRUE or FLASE

A. Smoking cessation can reduce the rates of exacerbations by 1/3

TOTAL SCORE: _____/2

True

B. Smoking cessation slows decline in FEV1 in patients with COPD

TOTAL SCORE: _____/2

True

Section 4:

Write admission orders for the patient presented in the clinical vignette:

Admit to medicine
Diet as tolerated
Activity as tolerated
Vital signs q8h (or more frequently), including oxygen saturation (SpO_2) (1 point)

CBC daily
Electrolytes, Creatinine daily (1 point)

Oxygen (NP or FM) to keep oxygen saturation 88-92% (1 point)

Antibiotics: Any of below **(1 point for drug name one point for dose)**
Azithromycin 500 mg iv od or 500mg followed by 250 mg po od x 5 days
Ceftriaxone 1g iv q24h
Cefuroxime 750 mg iv q8h or 500 mg po q12h
Septra (Cotrimoxazole) one tab ds po bid
Amoxicillin 500 mg po q8h
Doxycycline 100 mg po q12h
Clavulin (Amoxicillin an Clavulanic acid)
Moxifloxacin 400 mg iv or po q24h
Levofloxacin 500 mg po or iv od

Corticosteroids: Any of below **(1 point for drug name 1 point for dose)**
Prednisone (0.5-1 mg/kg) or (40-60mg) once daily
Methylprednisolone (Solu-medrol) 60-125mg frequency twice daily to four times daily (bid to qid)

Bronchodilators: Both required **(0.5 point for each drug name 0.5 point for each dose)**
Salbutamol at least 2 puffs at least q4h
Ipratropium at least 2 puffs at least q4h

DVT Prophylaxis: Any of below **(0.5 point)**
Heparin 5000u sc bid
Heparin 5000u sc tid
Heparin 7500u sc bid
Enoxaparin 40 mg sc od

Immunization
Any mention of immunization for influenza or pneumococcus **(0.5 point)**

TOTAL SCORE: _____/10
